# Catalytic Design of Matrix-Isolated Ni-Polymer Composites for Methane Catalytic Decomposition

**DOI:** 10.3390/polym15112534

**Published:** 2023-05-31

**Authors:** Mayya V. Kulikova, Mikhail I. Ivantsov, Anastasia E. Sotnikova, Vadim O. Samoilov

**Affiliations:** A.V. Topchiev Institute of Petrochemical Synthesis, Russian Academy of Sciences, Leninsky Prospect 29, 119991 Moscow, Russia

**Keywords:** catalytic design, Ni-polymer composite, polymer matrix, PVA, Ni/C catalysts, methane catalytic decomposition

## Abstract

Targeted synthesis of C/composite Ni-based material was carried out by the method of matrix isolation. The composite was formed with regard to the features of the reaction of catalytic decomposition of methane. The morphology and physicochemical properties of these materials have been characterized using a number of methods: elemental analysis, scanning electron microscopy (SEM), transmission electron microscopy (TEM), X-ray diffraction (XRD), Fourier transform infrared spectroscopy (FTIR), Raman spectroscopy, temperature programmed reduction (TPR-H_2_), specific surface areas (SSA), thermogravimetric analysis, and differential scanning calorimetry (TGA/DSC). It was shown by FTIR spectroscopy that nickel ions are immobilized on the polymer molecule of polyvinyl alcohol, and during heat treatment, polycondensation sites are formed on the surface of the polymer molecule. By the method of Raman spectroscopy, it was shown that already at a temperature of 250 °C, a developed conjugation system with sp2-hybridized carbon atoms begins to form. The SSA method shows that the formation of the composite material resulted in a matrix with a developed specific surface area of 20 to 214 m^2^/g. The XRD method shows that nanoparticles are essentially characterized by Ni, NiO reflexes. The composite material was established by microscopy methods to be a layered structure with uniformly distributed nickel-containing particles 5–10 nm in size. The XPS method determined that metallic nickel was present on the surface of the material. A high specific activity was found in the process of catalytic decomposition of methane—from 0.9 to 1.4 g_H2_/g_cat_/h, X_CH4_, from 33 to 45% at a reaction temperature of 750 °C without the stage of catalyst preliminary activation. During the reaction, the formation of multi-walled carbon nanotubes occurs.

## 1. Introduction

The rapid development of materials science has allowed for the creation of composite materials with “advanced” properties, which is particularly relevant for heterogeneous catalysis [1,2,3]. In this case, composite materials based on synthetic polymers can be considered as promising catalysts for many processes characterized by the required set of physicochemical characteristics [4,5,6].

One of the most important characteristics in the design of catalytically active materials is the requirement for the formation of a developed surface, high dispersion of the active phase, and a certain phase composition [7,8,9,10,11,12]. Modern materials science has developed various approaches for creating systems with a developed surface and dispersion [13,14,15]. Traditional methods for increasing the dispersion of active particles include the use of carriers with a highly developed surface and structural promoters for both the active phase (such as copper or cobalt) and the substrate used (La, Ce, Y) [16,17]. The structural promoters used affect both the phase composition and the oxide environment [18,19]. Nevertheless, the applied systems do not allow for the creation of highly concentrated (high-loaded) catalysts while maintaining a stable nanoscale state of the active component [20,21]. These limitations lead to the necessity of creating volumetrically structured highly dispersed catalytic systems primarily in the absence of any oxide carrier [22].

Composite materials can solve the problem of catalytic construction. The task can be partially solved by creating rigid frameworks based on nickel-containing foam [23].

Methods for limiting the mobility of nanoscale metal particles in a solid non-oxide carbon-based dispersion medium are more interesting [24,25]. This method of obtaining composites is called matrix isolation [26,27]. Varieties of this method include stabilizing the polymer suspension (microencapsulation) or forming distributed nanoparticles during thermal treatment of a solid joint solution of metal salt and polymer, which is accompanied by structuring and nanocapsulation of the metal-containing phase in a carbon matrix [28,29,30,31]. The nature of the reagent and the conditions of the catalytic reaction determine the methods of obtaining the composite [32,33].

Transition metals of group VIII are the basis of C1 chemistry processes. These processes are the basis of environmental technologies such as carbon capture and storage, hydrogen production, eco fuel production, and others [34,35].

The success of using C/composites for environmental processes is demonstrated in [36,37]. FT synthesis was carried out on composites prepared by the organic matrix method, and the synthesized materials showed high activity in the process of carbon monoxide hydrogenation; carbon monoxide conversion reached 100%, and the specific activity was 126 µmol_CO_/g_Fe_/g_Co_/s. It has been shown that samples with a narrow particle size distribution (d_avg_ from 5 to 14 nm) can be successfully synthesized by this method, which determines the catalytic activity of the sample due to the correlation of size and catalytic properties (up to 38 µmol/g_Fe_/g_Co_/s) [36].

In forming C/composite materials, there is often a system of polyconjugated bonds present that can interact with the active phase of the catalyst, altering the local electron density, which leads to improvements or deteriorations in catalytic properties. For example, Wang et al. [38] considered the case of the CO_2_ electroreduction reaction in the presence of Ni@C_3_N_4_ and Ni@C_3_N_4_-CN, and it is shown that in the case of Ni@C_3_N_4_, d-π conjugation occurs, which facilitates electron transfer from the metallic particle to the substrate, making it significantly difficult to activate CO_2_ molecules on the metal active sites. Ni et al. [39] examined catalysts for the electrochemical reduction of oxygen based on transition metals and conjugated polymers. The authors showed that strong d-π conjugation affects the angle between the d_xz_/d_yz_ orbitals of the centers formed by the transition metals, which leads to enhanced intermediate adsorption and amplifies the effect of electronic modulation of substituent groups on the ligands. The influence on the catalyst structure allows for control over the activity of the resulting catalytic systems and ultimately leads to the synthesis of a catalyst that surpasses the benchmark Pt/C. The effect of conjugated structures on catalytic properties is also shown in the case of photocatalysis [40]. Modification of TiO_2_ with compounds containing delocalized π-conjugated systems can increase catalytic activity, and the level of this enhancement can be modulated by various delocalized π-conjugated systems. Li et al. [41] examined nanosheets based on polymetalloporphyrins with a diene bond and possessing constant porosity and π-conjugation. When monodisperse nickel atoms are deposited on the nanosheets, a heterogeneous catalyst with unprecedented catalytic activity and selectivity for the reduction of 4-nitrophenol to 4-aminophenol under mild conditions is formed. According to the authors [42,43], in the case of the process of carbon monoxide hydrogenation, which forms the composite during the synthesis, the polyconjugated matrix that is formed interacts with the active metal-containing particles, promoting an enhancing effect and increasing the activity of composite catalysts.

One of the rapidly developing areas of catalysis is the catalytic decomposition of methane to obtain CO_x_-free hydrogen [44,45]. Hydrogen is at the forefront of new environmentally neutral technologies and can be used in fuel cells, energy, etc. [46,47,48,49,50]. The catalytic decomposition of methane is based on the following reaction [51]:

CH_4_ → C + 2H_2_ (1)

The catalysts used in this process can be divided into two classes: carbon-based [52] and metal-containing [44,53,54]. Carbon-based catalysts operate at higher temperatures, from 800 °C, while metal-containing catalysts work at 600–700 °C [55]. Combining both types of catalysts—carbon-based and metal-containing—seems promising. For example, the use of activated carbon (AC) in [56] increased the catalytic efficiency by acting as a co-catalyst and promoting the formation of catalytic carbon fibers.

Ni-PVA composites synthesized by the matrix isolation method through thermolysis in an inert atmosphere, which are the subject of this study, have not been described in the literature. Novell presents a synthesis of composite material based on polyvinyl alcohol, a water-soluble polymer with side oxygen-containing groups capable of exhibiting donor–acceptor interactions with active metal and thereby forming a highly dispersed catalytically active matrix with a predetermined phase composition. Ni-PVA composites were studied in the process of methane decomposition. This approach to composite synthesis is particularly relevant for these processes, as the active phase of the catalyst is the metallic nickel phase, which can be formed at the composite preparation stage. This is very relevant for industrial applications, as it potentially eliminates the activation stage of the catalytic system.

## 2. Materials and Methods

### 2.1. Materials

Nickel nitrate hexahydrate (analytical grade, Vekton, Moscow, Russia) and polyvinyl alcohol (reagent grade, Chang Chun Petrochemical Co., Ltd., Taipei, Taiwan), with a 16:1 ratio of hydroxyl groups to acetate groups, and distilled water were used without further purification.

### 2.2. Synthesis of Polymer–Metal Salt Precursor

In this work, catalysts were prepared as follows: polyvinyl alcohol was dissolved in distilled water under heating, and then an aqueous solution of nickel nitrate was gradually added while stirring. The solvent was removed by drying in a drying cabinet until a polymer film with a constant mass was obtained. The synthesized metal–polymer precursor after drying was a homogeneous and uniformly colored polymer film. The nickel content in the initial mixture of the polymer and salt corresponded to 20% by weight based on Ni^0^.

### 2.3. Thermal Treatment of the Precursor

The treatment was carried out under a nitrogen inert gas flow. The samples were kept at 100 °C for 30 min to remove traces of the solvent. The precursor was then destructed to form the metal–carbon matrix composite at temperatures of 250 °C, 350 °C, 500 °C, and 700 °C for one hour in a tubular quartz furnace.

### 2.4. Elemental Analysis

The nickel content was determined by flame atomic absorption analysis using an AAnalyst 400 instrument (Perkin Elmer, Waltham, MA, USA). The matrix was removed by incineration in a muffle furnace at 550 °C. The residue was dissolved in concentrated nitric acid. CHN analysis was conducted by chromatography of oxide mixtures obtained after sample combustion with oxygen in a dynamic flash at ≈2000 °C using a Thermo Flash 2000 analyzer (Thermo Fisher Scientific, Heysham, UK). The detector was a katharometer, and the carrier gas was helium.

### 2.5. Transmission Electron Microscopy

Transmission electron microscopy investigation was carried out using a JEOL JEM 2100 electron microscope (JEOL, Tokyo, Japan) at an accelerating voltage of 200 kV. Prior to the deposition of copper grids coated with a Formvar film, composites were suspended in hexane at a concentration of 0.05 g per 10 mL of solvent. To plot particle size distribution, more than 100 particles on 5 images were analyzed.

### 2.6. Scanning Electron Microscopy

Scanning electron microscopy investigation was carried out using a Phenom XL G2 electron microscope (Thermo Fisher Scientific, Breda, The Netherlands) at an accelerating voltage of 21 kV.

### 2.7. X-ray

X-ray diffraction analysis was performed at room temperature using a Rigaku (Oume, Japan) Rotaflex RU-200. Cr-Kα radiation with Bragg–Brentano focusing was used. The particle size of the active phase was calculated according to the Scherrer equation. The average crystallite sizes were calculated using the Debye–Scherrer formula:

D = 0.9·λ/(B·cosθ)(2)

### 2.8. The Surface Area

The surface area was determined using an ASAP 2020 V4.00 analyzer (Micromeritics, Norcross, GA, USA). The adsorption temperature was −195.7 °C, and the adsorbate was nitrogen. The specific surface was calculated by the BET method, and diameter and pore volume by the BJH method.

### 2.9. TPR-H_2_

The catalysts’ reduction during linear heating with hydrogen absorption process monitoring was conducted in a flow quartz reactor (2 mm diameter) with temperature control using a thermocouple at atmospheric pressure in the temperature range from room temperature to 800 °C. The H_2_/Ar gas flow (H_2_ content—5% vol.) and the hydrogen content in the gas outlet stream were determined using a chromatograph Krystallux-4000 M. The weight of the catalyst was 30 mg.

### 2.10. IR Spectroscopy

The IR spectra were recorded by reflection using a HYPERION-2000 IR spectrometer (Bruker, Billerica, MA, USA) coupled to an IFS-66ν/s Bruker IR Fourier transducer (600–4000 cm^−1^).

### 2.11. Raman Spectroscopy

The Raman spectra were obtained using a confocal Raman microscope Senterra II (Bruker, Billerica, MA, USA). A laser with a wavelength of 532 nm and a power of 0.25 mW was used to excite the Raman scattering. The accumulation time was 1 sec, the number of repetitions was 200, objective 50×, diffraction grating 400 lines/mm, resolution 4 cm^−1^, aperture 50 × 1000 µm. Ten spectra from different selected areas were recorded for each sample. Spectral processing was carried out using the OPUS 8.5 software package (Bruker, Billerica, MA, USA).

### 2.12. TGA/DSC Test

The thermal behavior (mass changes and thermal effects) of the polymer–metal salt system was investigated using the TGA/DSC 3+ Mettler Toledo instrument (Greifensee, Switzerland). Measurements were carried out in 150 µL corundum crucibles in the temperature range from room temperature to 1000 °C at a heating rate of 10 °C/min under an inert gas atmosphere (nitrogen). The flow rate was 70 mL/min.

### 2.13. XPS Spectroscopy

The study of the composite material samples’ surface was carried out by X-ray photoelectron spectroscopy on an X-ray photoelectron spectrometer (Prevac, Rogow, Poland). An X-ray tube with AlKα radiation (1486.6 eV) was used as a source of ionizing radiation. Before loading into the spectrometer, the samples were ground in an agate mortar and applied to conductive carbon tape. To neutralize the charge of the sample during the experiments, an electron-ion charge compensation system was used. All peaks were calibrated versus the C 1s peak at 284.8 eV. The type of background was Shirley and during deconvolution, it was assumed that the total peak was the sum of Gaussian curves.

### 2.14. Catalyst Activity Test

Catalysts were tested without pre-reduced stage. The methane decomposition reaction was carried out in a quartz flow tube reactor at 500–900 °C, 0.1 MPa, 99.9% vol. CH_4_, and GHSV of 1500 h^−1^. The temperature was increased stepwise (50 °C/30 min). The gas products were analyzed twice at each isothermal regime. A total of 200 mg of catalyst was loaded into the reactor.

### 2.15. Gas Analysis

The feed synthesis gas and gas products were analyzed by gas-solid chromatography on a Kristallyuks-4000 (Meta-chrom, Yoshkar-Ola, Russia) chromatograph equipped with a thermal conductivity detector using helium as a carrier gas. Two chromatographic columns were used. Carbon monoxide, nitrogen, and hydrogen were separated on a 3 m × 3 mm column packed with CaA molecular sieves in an isothermal mode at 80 °C; CO_2_ and C_1_-C_4_ hydrocarbons were separated on a HayeSep R-packed column (3 m × 3 mm) with temperature programming of 80 to 200 °C at 8 °C/min.

## 3. Results

### 3.1. IR Spectroscopy

**Figure 1:**
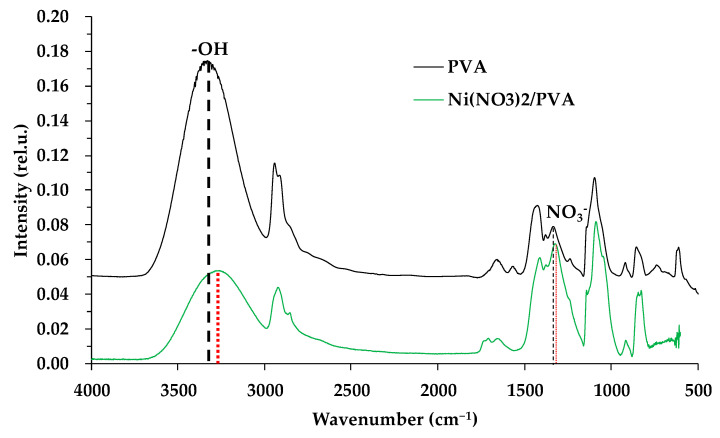
IR Fourier spectroscopy of samples of the original PVA and a mixture of {PVA + Ni(NO_3_)_2_}.

The formation of new chemical bonds during the composite preparation was monitored by Fourier transform infrared spectroscopy (Figure 1). The figure shows the spectra of the original PVA and the film obtained after evaporating the PVA and nickel nitrate solution at the first stage of the composite preparation.

**Figure 2:**
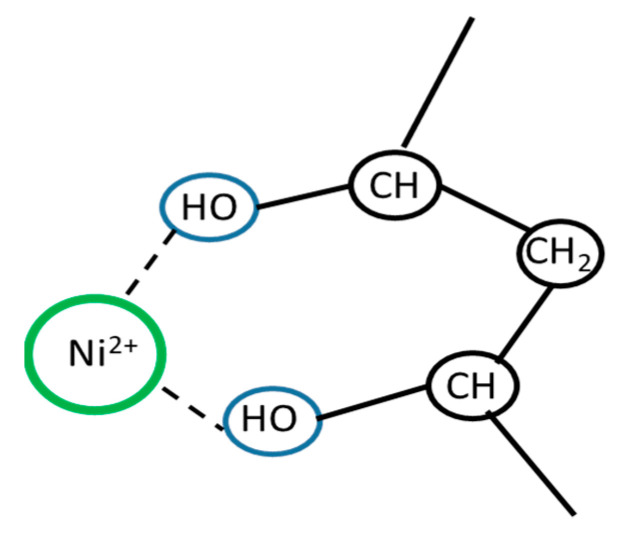
Structure of the fragment formed during the interaction of Ni^2+^ and polyvinyl alcohol.

The absorption bands characteristic of the hydroxyl group vibrations (maximum absorption band at 3336 cm^−1^ for PVA [57] and maximum absorption band at 3272 cm^−1^ for the mixture) are shifted to longer wavelengths. The formation of complex bonds occurs, in which the PVA molecule acts as a polydentate ligand [58,59]. A shift in the vibrations of the NO_3_^−^ group (1334 cm^−1^ to longer wavelengths) is also observed, indicating the formation of chemical bonds between the nickel ions and the polymer [60]. The interaction of nickel ions with the polymer molecule can be called the first stage of composite material formation. During the preparation stage of the joint metal–polymer system, distribution and immobilization of nickel ions occur on the macromolecule. Probably, the formation of a chelating complex compound occurs (Figure 2).

**Figure 3:**
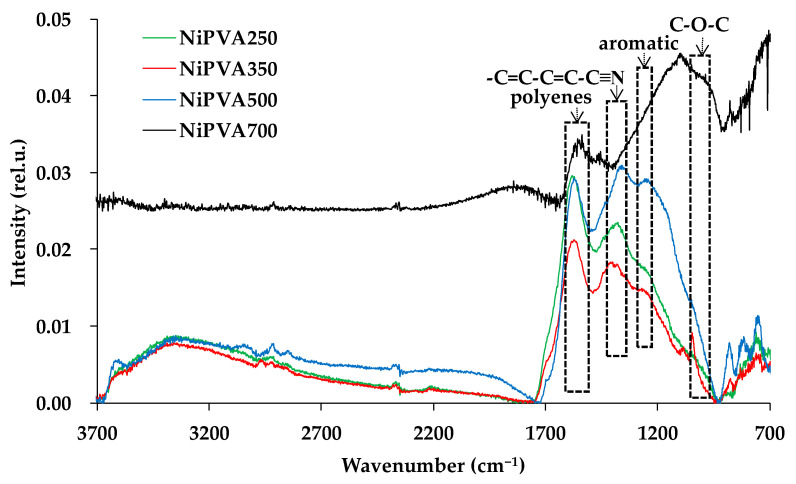
IR Fourier spectroscopy of composite materials obtained by thermal decomposition of a mixture of nickel nitrate and polyvinyl alcohol.

In Figure 3, IR spectra of composite materials formed at different temperatures are shown.

In the spectra of composite materials, characteristic bands of the polyconjugated fragments of the macromolecule are observed [61,62]. A shift in the absorption band at 1643 cm^−1^, characteristic of polyene hydrocarbon absorption [63], is recorded, indicating changes in both the length and composition of the conjugated fragments depending on the temperature of synthesis of the composite material. A broad band in the range of 1000–1100 cm^−1^ may indicate the formation of C-O-C and =C-O-C bonds, indicating the inclusion of oxygen in the composition of the conjugated fragments [64,65]. It is also possible to form conjugated fragments of the structure -C=C-C=C-C≡N (absorption bands in the range of 1370–1420 cm^−1^) and the formation of aromatic fragments (absorption bands at 1245–1270 cm^−1^) [66].

### 3.2. Raman Spectroscopy

**Figure 4:**
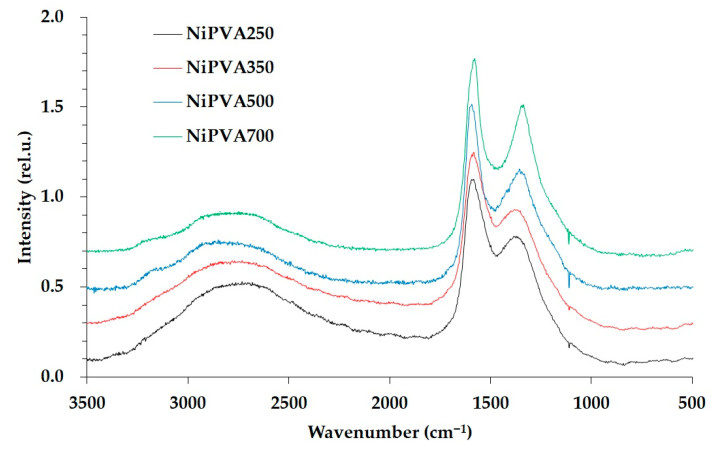
Raman spectra of Ni/PVA samples obtained at different thermal treatment temperatures.

In Figure 4, Raman spectra of composite materials formed at different temperatures are shown.

Raman spectra of Ni/PVA samples at 250 °C, 350 °C, and 500 °C exhibit a high-intensity band in the first-order region with peaks at 1370–1380 cm^−1^ (D-band) and 1581–1587 cm^−1^ (G-band). The G-band corresponds to valence vibrations of sp^2^ carbon atoms (C=C) in aromatic rings and carbon chains. The D-band corresponds to defects in the graphitic structure (e.g., non-hexagonal rings, sp^3^-hybridized carbon atoms) [67]. The second-order region contains unresolved low-intensity bands in the 3400–2200 cm^−1^ range, which represent overtones of the G and D bands and their combinations, typical of weakly ordered amorphous carbon [68]. The D/G intensity ratio is 0.69–0.72 at 250 °C, 0.64–0.75 at 350 °C, 0.61–0.67 at 500 °C, and 0.76–0.78 at 700 °C.

Thus, the formation of a developed conjugation system with sp^2^-hybridized carbon atoms is already observed at a temperature of 250 °C. Increasing the synthesis temperature did not significantly affect the ratio of carbon with different degrees of hybridization, indicating that the stiffness of the composite frame did not increase significantly with increasing temperature. This is atypical of the graphitization process of polymeric materials with increasing temperature. This fact may be related to the presence of nanosized nickel particles and their interaction with the carbon frame as a structuring matrix. A similar effect has been described in [69,70] for the interaction of nanosized iron oxide particles with polymer molecules. In addition, there are studies that describe the effects of plasticization of polymers with metal salts [71].

### 3.3. TGA/DSC Test

**Figure 5:**
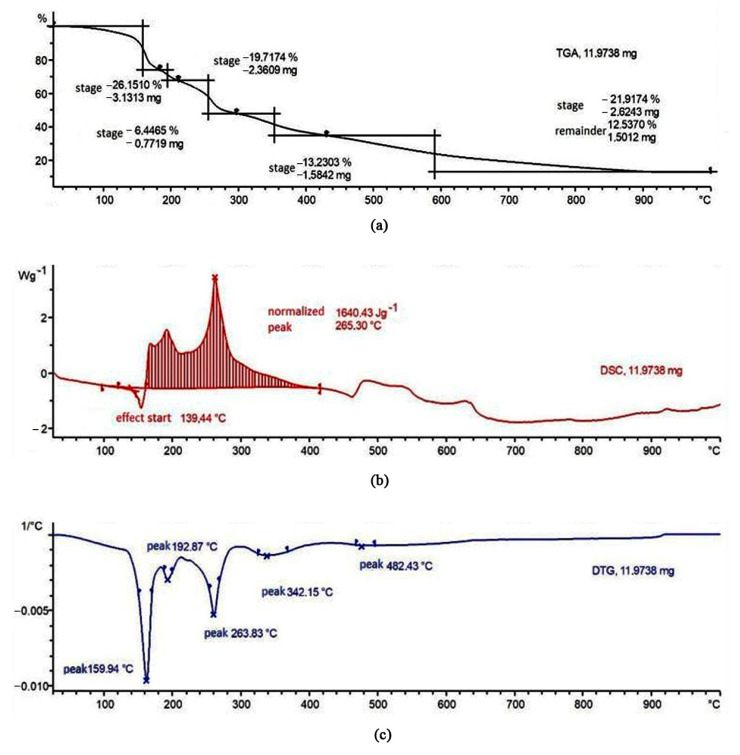
Thermal properties of composite precursor—a film of {PVA + Ni(NO_3_)_2_}: (**a**) thermal gravimetric analysis (TGA); (**b**) differential scanning calorimetry (DSC); (**c**) derivative thermogravimetry (DTG).

Figure 5 describes the study of thermal destruction of the precursor of the composite—a film. It is evident that during the formation of the film, there is an interaction between nickel ions and the polymer (Figure 1). In this case, it was interesting to investigate whether there would be any synergistic effects from the interaction of nickel ions and the polymer during thermal treatment.

According to the data described in [72], the thermal destruction of PVA without additives occurs through a series of sequential stages. In the 200–300 °C range, dehydration occurs with the formation of isolated double bonds (stage 1), and upon further heating to 350–400 °C, complete dehydration occurs with the formation of linear polyene conjugated structures (stage 2). Higher-temperature treatment at 400–450 °C leads to the cracking of linear polyenes and the formation of polyaromatic conjugated structures (carbonization of PVA) [73,74]. The decomposition of pure nickel nitrate hexahydrate also occurs stepwise [75]. Partial removal of water from the crystal hydrate is observed at 160 °C, and hydrolysis of the salt with removal of water and nitrate ions to form nickel hydroxysalts occurs at 190 °C. Complete decomposition to nickel oxide (II) occurs at temperatures above 270 °C.

As shown in Figure 5, when nickel nitrate is added, the main stages of decomposition of both PVA and the nitrate are also observed, but the characteristic temperatures of the stages related to the destruction of PVA are lowered. According to the authors, the peak in the T = 263 °C range during the film’s destruction corresponds to stage 1, dehydration of polyvinyl alcohol, and the peak in the 342 °C range corresponds to stage 2, dehydration with the formation of linear polyene conjugated structures.

This indicates that the precursor of the composite, the film, undergoes significant synergistic effects, indicating the interaction between nickel and the polymer, which correlates with the data from IR spectroscopy.

### 3.4. Elemental Analysis

The elemental composition of each composite obtained at different temperatures is presented in Table 1.

The same increase in carbon content with the increase in the synthesis temperature of the composite from 250 to 350 °C indicates the processes of dehydration (stage 1, described in Section 3.3), as well as a decrease in the proportion of heteroatoms. It is obvious that with the increase in the synthesis temperature of the composite, processes associated with the decomposition of the polymer, decomposition of the metal salt, and reduction by the products of decomposition of the inorganic component occur.

### 3.5. Morphology Characterization

**Figure 6:**
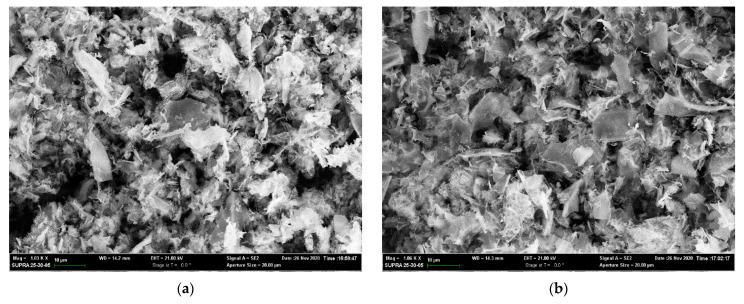

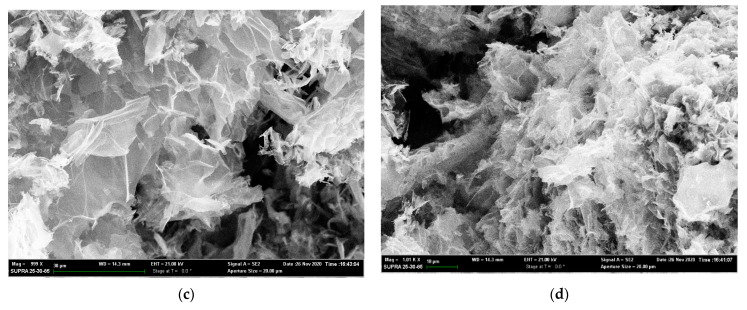
Scanning electron microscopy of (**a**) NiPVA250; (**b**) NiPVA350; (**c**) NiPVA500; (**d**) NiPVA700.

The morphology of the synthesized catalysts is presented in Figure 6.

The SEM results show that during processing, the dense structure is destroyed, forming a highly porous layered structure over the entire temperature range. Surface development is likely due to water removal at 160–190 °C and partial oxidation of the polymer by decomposition products of the inorganic component at 190 °C. The development of texture characteristics during thermal treatment is presented in Table 2.

Analysis of the results of porometry leads to the conclusion that thermal treatment of the metal–polymer mixture results in the formation of a composite with a developed surface. The most intense formation of pores occurs at temperatures exceeding 250 °C, which is consistent with thermal analysis data. Thus, the low specific surface area value for the sample treated at 250 °C is related to the initial formation of a dense carbon-containing matrix of partially pyrolyzed polymer. The optimal temperature for synthesis of composite material for catalysis can be considered to be 500 °C, since in this temperature range, the material with the most developed surface is formed. Further increase in processing temperature leads to a decrease in total specific surface area with a slight increase in pore diameter and volume, which may indicate matrix material densification by carbonization, resulting in the blocking of catalytic active sites. This is similar to the coating of a metal surface with carbon in high-temperature petrochemical processes.

**Figure 7:**
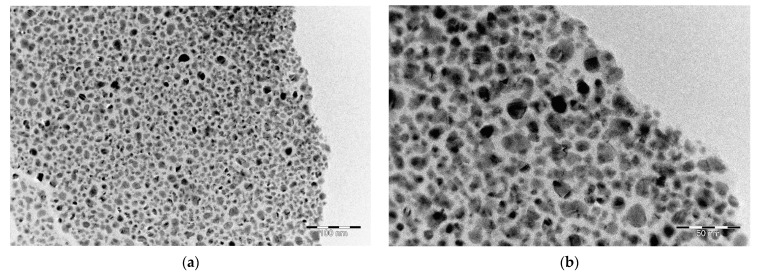
Transmission electron microscopy of NiPVA500 at different resolution: (**a**) 200 nm; (**b**) 50 nm.

Detailed investigation of the layers using TEM shows that the formed layers consist of homogeneously distributed metal-containing particles in a carbon matrix (Figure 7).

**Figure 8:**
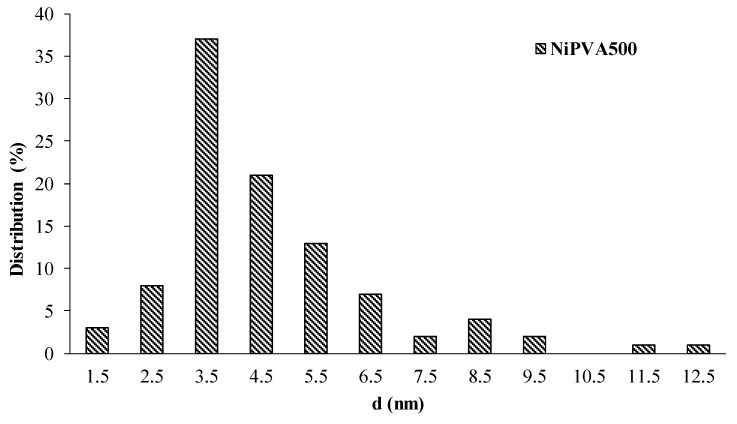
Size distribution of nickel-containing phase nanoparticles for sample of NiPVA500 composite material.

The distribution histogram (Figure 8) shows that most particles are in the range of 5–10 nm. There are a small number of particles of smaller and larger sizes, but no agglomerates were detected.

### 3.6. X-ray

**Figure 9:**
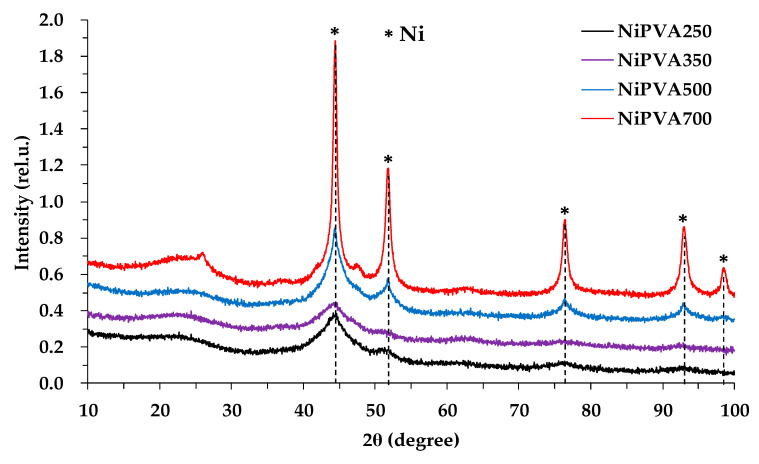
Diffractograms of composite materials.

Reflections of the crystalline phases Ni (PDF#65-2865), NiO (PDF#89-7130), and crystalline carbon (PDF#54-0501) were detected on the diffractograms of all samples (Figure 9). The average crystal size calculated by the Debye formula is presented in Table 3. Due to the large peak width, this characteristic is approximated by (111) crystal plane.

### 3.7. XPS

The quantitative composition of the material surface obtained at 500 °C is presented in Table 4. The surface concentrates such elements as carbon and oxygen (at 1.6 and 1.2 times, respectively), while the nickel content is reduced by a factor of 2.7 compared to the content in the bulk material, which may indicate partial encapsulation of the metallic particles. The nitrogen content remains practically the same, which may indicate a uniform distribution of nitrogen in the nitrogen-containing fragments at the stage of nitrate decomposition and polymer molecule nitration.

**Figure 10:**
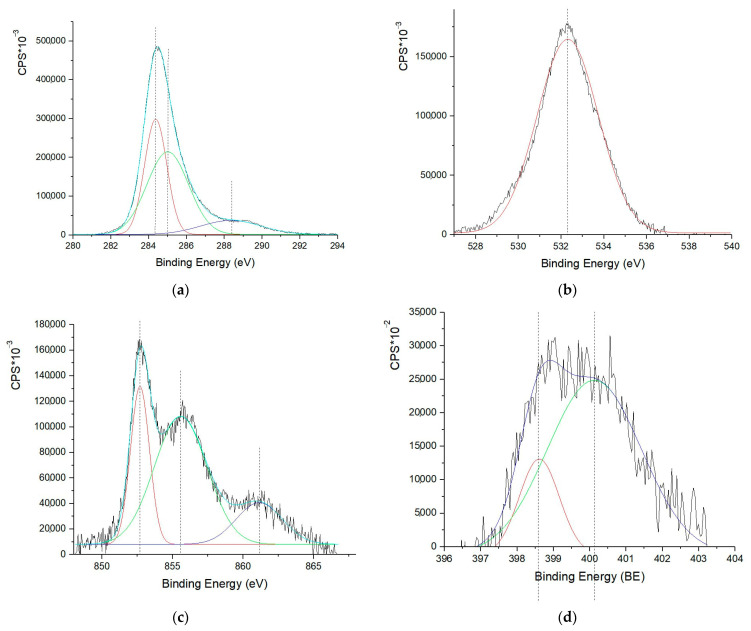
XPS spectra of elements: (**a**) C 1s; (**b**) O 1s; (**c**) Ni 2p3/2; (**d**) N 1s.

Based on the C 1s spectrum, carbon (Figure 10a) on the composite surface exists in three forms: C=C (sp^2^ -carbon) bonds (284.4 eV, FWHM 1.4 eV) [76], which account for about 58%, C-C (sp^3^ -carbon) bonds (285.0 eV, FWHM 2.6 eV) [77] at 30%, while 12% of carbon corresponds to carbon in acidic groups -C(O)-OH (288.4 eV, FWHM 3.7 eV), indicating the functionalization of the material surface [78]. Oxygen (Figure 10b) is present in functional groups C-O/C=O (532.3 eV, FWHM 3.4 eV) [79].

Deconvolution of the Ni 2p3/2 spectrum (Figure 10c) indicates that in addition to oxidized Ni present in the form of Ni-O or Ni(OH)_2_ (855.6 eV, FWHM 4.4 eV) with a content of 73%, metallic nickel (27%) (852.7 eV, FWHM 1.6 eV) [80,81] is also present. The spectrum also shows an intense peak at 861.2 eV (FWHM 3.9 eV), which corresponds to the satellite peak from the peak at 855.6 eV. Nitrogen (Figure 10d) is mainly (84%) found in amide and pyrrole functional groups (400.1 eV, FWHM 3.0 eV), while 16% is attributed to polyaniline or pyridine fragments (398.6 eV, FWHM 1.3 eV) [82,83].

### 3.8. TPR-H_2_

**Figure 11:**
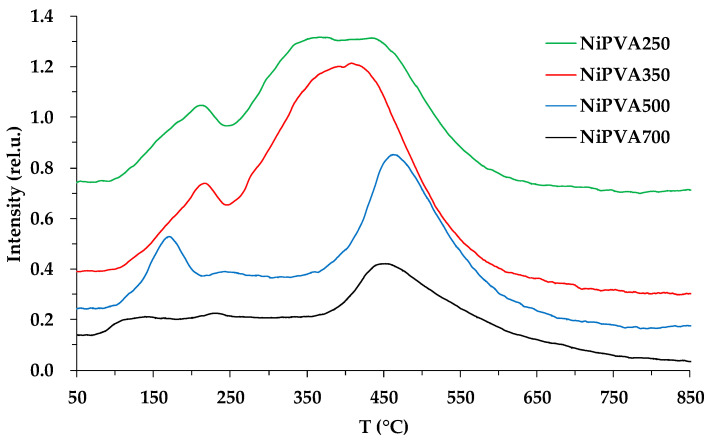
TPR profile of samples of composite materials.

The reducibility of the materials was examined by TPR measurements (Figure 11).

It should be noted that according to the XRD data, the NiO phase is practically absent in all samples. However, two characteristic regions, 170–220 °C and 410–470 °C, can be distinguished on the hydrogen absorption curves. Since the intensity of the first region significantly decreases with increasing treatment temperature and is practically absent in the NiPVA700 sample, we attribute it to matrix absorption, which removes oxygen and nitrogen atoms from the composite composition. The second temperature range corresponds well to the reduction in NiO weakly interacting with the carrier [84]. Moreover, the amount of absorbed hydrogen decreases with increasing treatment temperature, indicating the formation of a significantly X-ray-amorphous partially oxidized nickel phase at the initial stages. A similar effect was observed in the formation of a composite material based on cobalt. In situ magnetometry revealed that the formation of nanoparticles occurs by a diffusion mechanism and is associated with a gradual increase in the content of metallic cobalt from the nuclei of the new phase [85]. The authors attribute the increased width of the second peak to the encapsulation of nickel by an incompletely pyrolyzed matrix, during the decomposition and reduction of which highly active nickel becomes accessible.

### 3.9. Catalytic Activity

**Figure 12:**
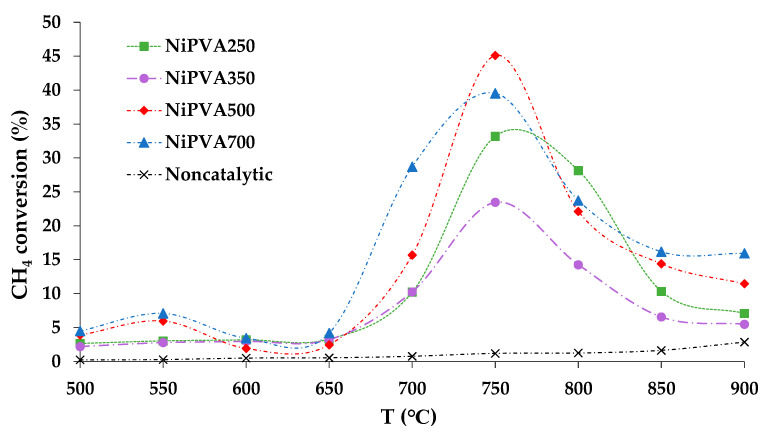
Temperature dependence of methane conversion in the thermocatalytic methane decomposition reaction for samples of composite materials.

The main feature of the synthesized Ni-PVA composites was that these catalytic materials exhibited activity in the thermocatalytic methane decomposition reaction without pre-reduction in a hydrogen stream. Widely described in the literature, oxide and deposited catalysts for this process [86,87,88,89,90] require pre-reduction to form the active phase of metallic nickel. This stage is high-temperature (450 °C and above) and requires the use of hydrogen as a raw material. The approach proposed by the authors in this work allows the formation of an active catalytic phase of metallic nickel at the synthesis stage of the composite, which is promising not only for catalytic design but also in the projection on the industrial use. A screening of catalytic properties was carried out beforehand, in which the main parameters of the methane catalytic decomposition process were compared in the temperature range from 500 °C to 900 °C; at each point the test was carried out for 30 min. The figure shows dependence of methane conversion on the experiment temperature (Figure 12).

The curves of the thermocatalytic decomposition of methane, shown in Figure 12, represent the classic bell-shaped characteristic curves [91,92]. Separately, it should be noted that the temperature range in which the catalysts exhibit high activity was 700–800 °C, which is slightly higher than the traditional one for Ni-containing catalysts [93]. It can also be noted that traditional carbon catalysts are active at higher temperatures, from 800 to 850 °C [94]. It can be assumed that this behavior of the composite catalyst is related to its “hybrid” nature, in particular with partial encapsulation of the active metal surface.

Figure 12 also shows the results of methane decomposition in the absence of the catalyst. Despite the fact that in the studied temperature range ∆G_r_ < 0 [91], the methane conversion is significantly lower than the results obtained in the presence of catalysts.

Based on the results of these tests, it was revealed that the highest methane conversion on all synthesized composites is typical for a temperature of 750 °C. The main parameters of the process at a temperature of 750 °C are shown in Table 5.

The activity of Ni-PVA composites prepared at different temperatures was 1.9–3.2 g_H2_/g_Ni_/h versus 0.4–1.8 g_H2_/g_Ni_/h (the given values were calculated based on research data) [44,95,96,97]. The specific activity of the catalysts predominantly was 0.15–0.39 mol_H2_/g_Cat_/h. A range of catalytic systems with prospects for methane thermocatalytic decomposition is described in the study by the authors of [98]. The activity of Ni composites prepared by direct catalytic construction shows a much higher yield of the target product—hydrogen—than all catalytic systems described in the study [80,81,82].

Additionally, it should be noted that Ni-based systems with promoters such as noble metals usually exhibit the highest activity [99,100,101]. Noble metals are traditionally added to deposited systems to reduce the temperature of preliminary reduction and intensify the hydrogen spillover process [102]. This effect was achieved in this study by directed construction of a catalytic system with a predetermined phase composition and nanoscale size of the active particles. Furthermore, a part of the catalytic surface is covered with a carbon matrix, which serves as a protective capsule and prevents oxidation of the active catalyst phase.

It is worth noting the extreme dependence of the activity of the composites on the temperature of their synthesis. Maximum activity was observed in the sample prepared at 500 °C. Such a dependence can be explained by the size effect and the degree of graphitization of the composite. For example, at a synthesis temperature of 700 °C, the largest nickel crystals with a size of 13 nm were detected. The mechanism of methane catalytic decomposition is described in detail in [103]. Obviously, a size-dependent catalytic effect is observed for this reaction. During the methane decomposition process and the growth of nanotubes, complex spatial structures are formed, which are difficult to form on small particles. This can explain the fixation of the maximum catalytic activity of the composite obtained at 500 °C with particle size of 4 nm.

**Figure 13:**
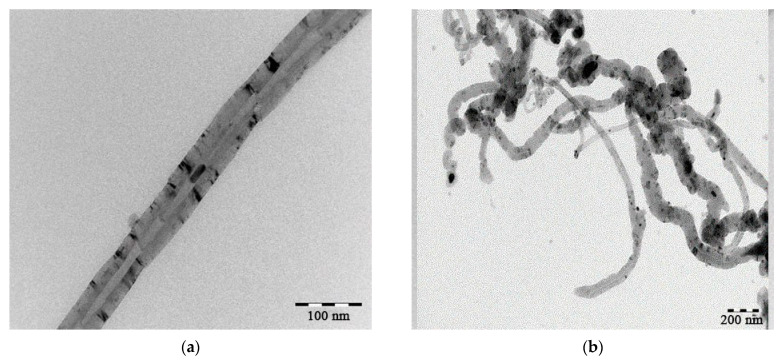
Carbon nanotubes (**a**,**b**) formed during methane decomposition.

It is known [104] that during the process of catalytic decomposition of methane, carbon of various structures can be formed. In the case of using Ni composites for this process, carbon nanotubes were formed, the microphotographs of which are presented in Figure 13.

## 4. Conclusions

Thus, in this work, a new C/composite material based on nickel and polyvinyl alcohol was synthesized and characterized, which was prepared by the organic matrix method. The composite synthesis was carried out with the directed formation of nanosized metallic nickel particles and the formation of a metallic nickel phase during the composite synthesis. The described approach made it possible to form nanosized metallic particles immobilized in a C-containing matrix with a narrow distribution of 5–15 nm. At the same time, some of the nickel-containing particles were partially encapsulated.

The choice of the phase composition and size of the metallic component crystal was carried out with projection on the methane decomposition reaction. The use of the directed construction method of the catalytic system gave a pronounced catalytic effect:

1. Elimination of the first stage of the catalyst’s reducing activation;
2. Sharp increase in the activity of the nickel-containing composite in terms of the sorption of reagents and the yield of the target product, hydrogen;
3. Further detailed study of the process of nanotube growth on the described composite material is of interest. It is possible that the process of nanotube growth in this case will proceed along routes different from the growth of nanotubes by the mechanism of carbon diffusion [103]. The partial carbon encapsulation of nickel particles in the composite material provides evidence for this assumption. It is possible that surface carbon may serve as a precursor for the nucleation of a carbon nanotube, the growth of which may occur by incorporating carbon fragments specifically on this catalytic surface.

The effects obtained provide a whole range of opportunities for further study of the properties and characteristics of such composite materials. The formation of metal-containing composites with a narrow distribution of metallic particles is an extremely promising approach for synthesizing catalysts for structurally sensitive processes and processes with pronounced diffusion limitations.

## Figures and Tables

**Table 1:** Texture characteristics depending on the temperature of composite material formation.

Sample	Synthesis Temperature, °C	Ni Content, wt. %	C Content, wt. %	N Content, wt. %	H Content, wt. %	O Content, wt. %
NiPVA250	250	31	39	2	2	26
NiPVA350	350	43	39	2	2	14
NiPVA500	500	43	42	2	1	12
NiPVA700	700	48	44	1	1	6

**Table 2:** Texture characteristics depending on the temperature of composite material formation.

Sample	S_sp_, m^2^/g	V_pore_, mL/g	D_a_, nm
NiPVA250	21	0.02	4.0
NiPVA350	158	0.09	2.4
NiPVA500	214	0.10	2.0
NiPVA700	204	0.11	2.2

**Table 3:** Crystallite size characteristics depending on the temperature of composite material formation.

Sample	Crystallite Size, nm
NiPVA250	2.5–3.0
NiPVA350	2.5–3.0
NiPVA500	4.0
NiPVA700	13.0

**Table 4:** Quantitative composition of the sample surface of composite material obtained at 500 °C.

Sample	Ni	O	C	N
Content, % at.	4	13	81	2
Content, % wt.	16	14	67	2

**Table 5:** Main indicators of the catalytic decomposition of methane at a temperature of 750 °C in the presence of composite catalysts based on polyvinyl alcohol and nickel nitrate.

Samples	X_CH4_, %	A, g_H2_/gCat/h	Yield, g_H2_/g_Ni_/h	Feed, g_CH4_/gCat/h
NiPVA250	33	0.9	3.0	24.1
NiPVA350	38	1.4	3.2	23.8
NiPVA500	45	1.3	3.1	11.9
NiPVA700	40	0.9	1.9	9.1

## Data Availability

Not applicable.

## Nomenclature/Abbreviations

SEM	Scanning electron microscopy
TEM	Transmission electron microscopy
XRD	X-ray diffraction
FTIR	Fourier transform infrared spectroscopy
TPR-H_2_	Temperature programed reduction
SSA	Specific surface areas
TGA/DSC	Thermogravimetric analysis and differential scanning calorimetry
FT synthesis	Fischer-Tropsch synthesis
PVA	Polyvinyl alcohol
Ni-PVA	Composites synthesized by the matrix isolation method
PVA + Ni(NO_3_)_2_	The film obtained after evaporating of the polyvinyl alcohol and nickel nitrate joined solution
GHSV	Gas hourly space velocity, h^−1^
S_sp_	Surface area, m^2^/g
V_pore_	Total pore volume, ml/g
D_a_	Pore size, nm
D	Crystallite sizes, nm
λ	Wavelength, nm
B	Full width at half maximum
θ	Diffraction angle, °
∆G_r_	Gibbs energy, kJ/mol
X_CH4_	Methane conversion, %
A	Activity, g_H2_/g_Cat_/h
